# Supplementary material for: Dynamic transcriptomic profiles of zebrafish gills in response to zinc supplementation
Source: BMC Genomics. 2010 Oct 11;11:553. doi: 10.1186/1471-2164-11-553 (PMC3091702; doi:10.1186/1471-2164-11-553)
Supplement: Additional file 2 — Interactive Direct Interaction Network representing the molecular interactions between zinc, copper, iron, calcium and proteins encoded by transcripts changed by zinc supplementation. Mini web-site containing index.html and hyperlinked pages in subdirectory describing a Direct Interaction Network automatically generated based on curated interactions contained within the proprietary PathwayArchitect database. Ovals represent proteins and the circles symbolize metal ions. Objects are coloured by their abundance in zebrafish at the time-point they were significantly different from the control is a scale from -4 fold (dark green) to +4 fold (dark red). Where significant differences were found at more than one time-point, the colour overlay shows expression at the first instance. Dark blue squares denote 'binding', and light blue squares 'expression'; green squares stand for 'regulation', green diamonds for 'metabolism', and green circles for 'promoter binding'. Arrow heads indicate directionality of the interaction where annotated. All nodes and edges can be further interrogated by selecting the relative area of the image. [file 1471-2164-11-553-S2.zip › PathwayArchitect Zn xs DIN/640496.html]

# REGULATION:

|  |  |
| --- | --- |
| Type | REGULATION |
| Effect | Positive |


---

|  |  |
| --- | --- |
| Score | 0 |


---

|  |  |
| --- | --- |
| Reference Count | 14 |


---

|  |  |
| --- | --- |
| Mechanism | Unknown |


---

|  |  |
| --- | --- |
| Reference:0 || Sentence | "These results indicate that zinc contents in liver and in metallothionein did not increase in response to copper injection." |
| PMID | 9029672 |
| Year | 1996 |
| Species | Rat |
| Journal | Res Commun Mol Pathol Pharmacol |
| RefScore | 1 |
| Source | PArchNLP |
  |
|  |  |


---

|  |  |
| --- | --- |
 Reference:1 || Sentence | "Treatment of Saccharomyces cerevisiae cells with copper induces the activity of Cu/Zn superoxide dismutase (SOD) and catalase." |
| PMID | 8299153 |
| Year | 1993 |
| Journal | Curr Genet |
| RefScore | 1 |
| Source | PArchNLP |
  ||  |  |


---

|  |  |
| --- | --- |
 Reference:2 || Sentence | "Seven subjects (6.5%) fell below the criteria for low serum zinc levels (< 11.5 mumol.l-1) while two subjects (1.8%) were below the criteria for low serum calcium levels (< 2.20 mmol.l-1). 2) Therapeutic oral iron supplementation was successful in raising mean serum ferritin values from 15.9 micrograms.l-1 to 36.5 micrograms.l-1 but was not associated with decrements in serum copper or calcium levels. 3) The treatment did not significantly effect serum zinc and magnesium levels during the supplementation period, but a downward trend continued through the discontinuation phase so that at 18 and 24 wk serum zinc and magnesium levels were significantly lower than baseline. 4) Oral contraceptive use was associated with elevated serum copper and ferritin values and lowered serum magnesium levels." |
| PMID | 8492683 |
| Year | 1993 |
| Species | Human |
| Journal | Med Sci Sports Exerc |
| RefScore | 4 |
| Source | PArchNLP |
  ||  |  |


---

|  |  |
| --- | --- |
 Reference:3 || Sentence | "The effects of continuous exposure to cadmium (Cd) and ethanol on Cd turnover and zinc (Zn) and copper (Cu) body status of male Wistar rats were studied." |
| PMID | 12003907 |
| Year | 2002 |
| Species | Rat |
| Journal | Alcohol Alcohol |
| RefScore | 1 |
| Source | PArchNLP |
  ||  |  |


---

|  |  |
| --- | --- |
 Reference:4 || Sentence | "The present study was conducted to assess in rats the effects of oral aluminum (Al) exposure on calcium (Ca), magnesium (Mg), manganese (Mn), copper (Cu), zinc (Zn), and iron (Fe) accumulation and urinary excretion." |
| PMID | 11318238 |
| Year | 2001 |
| Species | Rat |
| Journal | Biol Trace Elem Res |
| RefScore | 1 |
| Source | PArchNLP |
  ||  |  |


---

|  |  |
| --- | --- |
 Reference:5 || Sentence | "On the contrary, cortical neurons expressing human copper-zinc superoxide dismutase exhibited increased apoptotic nuclei in both untreated and amyloid beta-peptide(25-35)-exposed neurons." |
| PMID | 10426499 |
| Year | 1999 |
| Species | Mouse |
|  | Human |
| Journal | Neuroscience |
| RefScore | 0 |
| Source | PArchNLP |
  ||  |  |


---

|  |  |
| --- | --- |
 Reference:6 || Sentence | "While hCCS can bind several copper ions, metal binding studies in the presence of excess copper scavengers that mimic the intracellular chelation capacity indicate a limiting stoichiometry of one copper and one zinc per hCCS monomer." |
| PMID | 11018045 |
| Year | 2000 |
| Species | Human |
| Journal | J Biol Chem |
| RefScore | 1 |
| Source | PArchNLP |
  ||  |  |


---

|  |  |
| --- | --- |
 Reference:7 || Sentence | "This paper describes modulation of these increases by copper intake, and describes combined effects of inflammation and copper intake on Cu-Zn and extracellular (EC) superoxide dismutase (SOD) activities." |
| PMID | 2560608 |
| Year | 1989 |
| Species | Human |
|  | Rat |
| Journal | Adv Exp Med Biol |
| RefScore | 1 |
| Source | PArchNLP |
  ||  |  |


---

|  |  |
| --- | --- |
 Reference:8 || Sentence | "The new ligands were highly efficient in the copper-catalyzed conjugate addition of Et(2)Zn to enones with up to 98% enantiomeric excess." |
| PMID | 12585909 |
| Year | 2003 |
| Journal | J Org Chem |
| RefScore | 0 |
| Source | PArchNLP |
  ||  |  |


---

|  |  |
| --- | --- |
 Reference:9 || Sentence | "The beneficial effects of S-adenosyl-L-methionine (SAM) in potentiating the mobilization of cadmium by cadmium trisodium diethylenetriamine penta acetic acid (DTPA) from the major target organs and restoration of depleted tissue glutathione (GSH), zinc and copper concentration, were determined in cadmium-exposed mice." |
| PMID | 11258544 |
| Year | 2001 |
| Species | Mouse |
| Journal | Cytobios |
| RefScore | 1 |
| Source | PArchNLP |
  ||  |  |


---

|  |  |
| --- | --- |
 Reference:10 || Sentence | "These results indicate that zinc binding to histatin 5 involves His-15 present within the -H-E-X-X-H- zinc binding motif, and copper binding occurs within the N-terminal D-S-H-, ATCUN motif." |
| PMID | 11226423 |
| Year | 2001 |
| Species | Human |
| Journal | FEBS Lett |
| RefScore | 3 |
| Source | PArchNLP |
  ||  |  |


---

|  |  |
| --- | --- |
 Reference:11 || Sentence | "The effect of cadmium, zinc, and copper accumulation on metallothionein content in the selected tissues of bank voles and yellow-necked mice trapped near the Sendzimir Steelworks in Krakow and the zinc smelter in Bukowno were analysed." |
| PMID | 12729168 |
| Year | 2002 |
| Species | Mouse |
| Journal | Folia Biol (Krakow) |
| RefScore | 1 |
| Source | PArchNLP |
  ||  |  |


---

|  |  |
| --- | --- |
 Reference:12 || Sentence | "Zinc's mechanism of action involves induction of intestinal cell metallothionein (Mt), which blocks copper absorption from the intestinal track." |
| PMID | 11585025 |
| Year | 2001 |
| Species | Human |
| Journal | Expert Opin Pharmacother |
| RefScore | 0 |
| Source | PArchNLP |
  ||  |  |


---

|  |  |
| --- | --- |
 Reference:13 || Sentence | "Injection of zinc and also of cadmium and copper had the opposite effect and increased MT-I concentrations in both samples, although the effects of zinc on blood cells and urine were relatively transient." |
| PMID | 3309208 |
| Year | 1987 |
| Species | Rat |
| Journal | J Nutr |
| RefScore | 2 |
| Source | PArchNLP |
  |


---

|  |  |
| --- | --- |
